# Total Neoadjuvant Therapy, Organ Preservation and Beyond: A State-of-the-Art Systematic Review and Critical Appraisal of Locally Advanced Rectal Cancer Management

**DOI:** 10.3390/diseases14050182

**Published:** 2026-05-21

**Authors:** Nabil Ismaili

**Affiliations:** 1 Department of Medical Oncology, Mohammed VI Faculty of Medicine, Mohammed VI University of Sciences and Health (UM6SS), Casablanca 82403, Morocco; ismailinabil@yahoo.fr or nismaili@um6ss.ma; 2 Mohammed VI Foundation of Sciences and Health (FM6SS), Casablanca 82403, Morocco; 3 Oncopathology, Biology and Environment of Cancer Laboratory, Mohammed VI Center of Research and Innovation (CM6RI), Rabat 11103, Morocco

**Keywords:** rectal cancer, total neoadjuvant therapy, chemoradiotherapy, organ preservation, watch-and-wait, immunotherapy, predictive factors, ctDNA, MRI-LINAC

## Abstract

Background: Locally advanced rectal cancer (LARC) management has evolved, but surgery (total mesorectal excision, TME) remains the curative cornerstone. Total neoadjuvant therapy (TNT) and organ preservation (OP) have emerged as response-adaptive strategies. We conducted a state-of-the-art systematic review to critically appraise TNT efficacy, trade-offs, OP feasibility, and emerging biomarkers. Methods: Following PRISMA 2020 guidelines, we searched PubMed, MEDLINE, Scopus, and EMBASE (1990–March 2026) plus ASCO/ESMO abstracts (2020–2026). We included phase II/III randomised controlled trials and major prospective studies evaluating neoadjuvant strategies in non-metastatic LARC. Risk of bias was assessed using RoB 2. Given heterogeneity, a narrative synthesis was performed (PROSPERO: CRD420251252675). Results: From 2847 records, 45 publications (30 trials) were included. For high-risk LARC (cT4, cN2, EMVI+, MRF+, tumour deposits), TNT improves disease-free survival and reduces distant metastases versus standard chemoradiotherapy (RAPIDO, PRODIGE 23, STELLAR, TNTCRT). However, TNT increases locoregional recurrence risk with short-course radiotherapy (RAPIDO: 10% vs. 6%; Polish II: no sustained overall survival benefit). Organ preservation is achievable in expert centres (OPRA: 54% 5-year TME-free survival; OPERA; CAO/ARO/AIO-16), but surgery remains the durable standard for most patients. De-escalation (PROSPECT, CONVERT, FOWARC, OCUM) avoids radiotherapy in low-risk (mrMRF−) patients without compromising local control. Lateral pelvic lymph node involvement (LPLN+) remains a negative prognostic factor even after TNT. Immunotherapy added to TNT (UNION, STELLAR II, SPRING-01, PRECAM) increases pCR rates (40–60%) but remains investigational. ctDNA-guided adaptation (CINTS-R) is feasible but requires mature data. Conclusions: Surgery (TME) is the definitive curative treatment for LARC. TNT is a preferred intensification strategy for high-risk patients, but trade-offs between systemic and local control must be individualised. Organ preservation is safe only for selected patients in expert centres. Immunotherapy-TNT combinations and ctDNA guidance are promising but not yet standard. This review provides an evidence-based roadmap for integrating these advances without losing sight of surgery’s central role.

## 1. Introduction

Rectal cancer represents a significant global health challenge. With colorectal cancer ranking as the third most common cancer and second leading cause of cancer mortality worldwide (1.9 million new cases, 904,000 deaths in 2022), rectal malignancies account for approximately one-third of this burden [1]. The management of locally advanced rectal cancer (LARC), encompassing stage II (T3–4, N0) and III (any T, N+) disease, has undergone a revolutionary transformation over the past three decades. The historical standard of surgery alone yielded unacceptably high rates of local recurrence and poor survival [2]. The sequential introduction of total mesorectal excision (TME), preoperative chemoradiotherapy (CRT), and adjuvant chemotherapy progressively improved outcomes, establishing a multimodal paradigm [3,4,5,6,7].

Despite these advances, distant metastasis remained the primary cause of death, highlighting the need for more effective systemic therapy. This led to total neoadjuvant therapy (TNT), which administers all planned chemotherapy before surgery [8]. TNT aims to target micrometastatic disease earlier, improve treatment compliance, and enhance tumor downstaging [8,9]. Concurrently, the pivotal observation that patients achieving a clinical complete response (CCR) after neoadjuvant therapy could be safely managed with a non-operative “watch-and-wait” (WW) strategy established the foundation for organ preservation (OP), shifting a core therapeutic goal from mandatory radical resection to selective, response-adapted management [10].

Landmark phase III trials, including RAPIDO, PRODIGE 23, and STELLAR, have provided Level I evidence establishing the superiority of TNT over standard CRT for high-risk LARC [11,12,13]. The TNTCRT trial further confirmed the benefit of LCRT-based TNT with CAPOX, showing a 3-year DFS of 77.0% vs. 67.9% (HR 0.623, *p* = 0.009) and a pCR rate of 27.5% vs. 9.9% [14]. Prospective studies like OPRA and OPERA have furnished robust Level II evidence supporting the oncologic safety and feasibility of OP [15,16,17]. A second wave of innovation is characterized by: (1) the successful integration of immunotherapy into TNT for MSS disease (UNION, STELLAR II, SPRING-01, PRECAM) [18,19,20,21,22]; (2) technological advances in radiotherapy delivery, such as proton therapy (PRORECT), magnetic resonance imaging (MRI)-guided radiotherapy (MRI-LINAC), and contact brachytherapy boosts (OPERA) [16,23,24]; and (3) the refinement of risk stratification through predictive biomarkers (Immunoscore biopsy), circulating tumor DNA (ctDNA) as in the CINTS-R trial and nuanced clinical response grading [25,26].

This rapidly evolving landscape presents new complexities and critical areas requiring further definition, including the optimization of chemotherapy and radiotherapy sequencing within TNT, the establishment of reliable criteria for CCR and the standardized grading of near-complete responses (NCR), the identification of ideal candidates for OP via biomarkers, delineation of the role of novel radiotherapy modalities in toxicity mitigation, and the development of tailored management strategies for challenging scenarios such as lateral pelvic lymph node involvement (LPLN+) and the growing population of elderly, potentially frail patients [9,27,28,29].

Furthermore, the optimal chemotherapy backbone within TNT remains debated. The MONT-R trial compared CapeOX versus capecitabine alone during neoadjuvant chemoradiotherapy in high-risk LARC, demonstrating that adding oxaliplatin significantly improved tumor regression (CAP 0–1: 58.6% vs. 46.8%; *p* = 0.011) but did not translate into a 3-year DFS or OS benefit, with comparable pCR rates (25.5% vs. 25.3%). Thus, oxaliplatin enhances local tumor regression, but its impact on long-term survival remains uncertain [30,31].

This systematic review provides a comprehensive, contemporary synthesis of the evidence for TNT and OP in LARC. We move beyond reporting aggregate efficacy to dissect predictive factors for treatment response, analyze the impact of technological innovations, and offer evidence-based perspectives on managing specific clinical scenarios [9,31,32,33,34,35,36,37,38,39]. By integrating data from over 30 pivotal trials and their key ancillary analyses, this review serves as a definitive guide for clinicians and a roadmap for future research.

## 2. Materials and Methods

### 2.1. Search Strategy and Study Selection

A systematic literature search was conducted in accordance with the Preferred Reporting Items for Systematic Reviews and Meta-Analyses (PRISMA) guidelines. Electronic databases (PubMed, MEDLINE, Scopus, EMBASE) were searched from January 1990 through March 2026. Manual searches of conference abstracts from the European Society for Medical Oncology (ESMO) and the American Society of Clinical Oncology (ASCO) (2020–2026) were also performed. The search strategy employed a combination of Medical Subject Headings (MeSH) terms and free-text keywords, including: “rectal neoplasms”/”rectal cancer”, “neoadjuvant therapy”, “chemoradiotherapy”, “total neoadjuvant therapy”, “immunotherapy”, “PD-1 inhibitor”, “organ preservation”, “watch-and-wait”, “non-operative management”, “contact x-ray brachytherapy”, “proton therapy”, “MRI-LINAC”, “ctDNA”, “quality of life”, “aged”/“elderly”, “lymph nodes, pelvic”.

### 2.2. Study Selection and Analytical Approach

**Figure 1:**
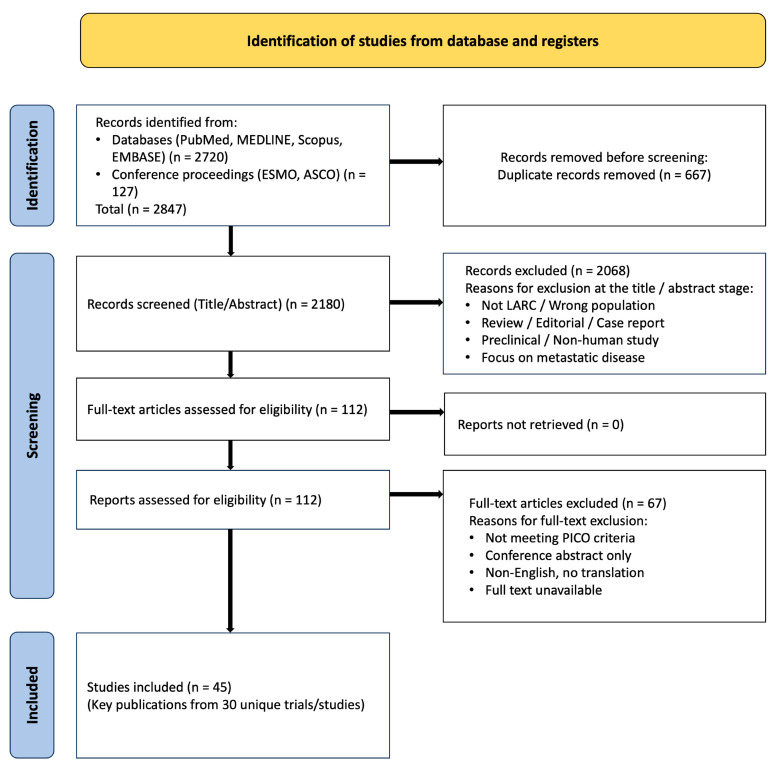
PRISMA flow diagram. The diagram illustrates the study identification, screening, eligibility, and inclusion process for the systematic review. LARC: Locally Advanced Rectal Cancer.

We included phase II/III randomized controlled trials and major prospective phase II studies evaluating neoadjuvant strategies in non-metastatic LARC. Given the significant clinical and methodological heterogeneity across studies, particularly in chemotherapy regimens, radiotherapy schedules, and the integration of novel agents, a formal quantitative meta-analysis was deemed inappropriate. This review is therefore a systematic narrative synthesis (a state-of-the-art review). While the literature search was systematic, the analysis is interpretive and thematic, aiming to synthesize evidence for clinical application rather than to produce a pooled quantitative estimate. The process is summarized in a PRISMA flow diagram (Table S1 and Figure 1).

### 2.3. Protocol Registration

Clinical trial number: Not applicable. Although this work follows a systematic search strategy, it is reported as a state-of-the-art expert review and was prospectively registered in PROSPERO for transparency. The study protocol was under registration number CRD420251252675.

### 2.4. Data Synthesis

Given the significant clinical and methodological heterogeneity across studies, particularly in the choice of chemotherapy regimens, radiotherapy protocols (short-course vs. long-course), sequencing (induction vs. consolidation), and the integration of novel agents (immunotherapy), a formal quantitative meta-analysis was deemed inappropriate. Therefore, a narrative synthesis was performed. Results are organized thematically by major treatment strategies and research questions, supported by structured summary tables to facilitate comparison and interpretation.

## 3. Results

### 3.1. Risk of Bias Assessment, Study Selection, and Characteristics

#### 3.1.1. Study Selection and Characteristics

The initial database search yielded 2847 records. After removal of duplicates and screening of titles/abstracts, 112 full-text articles were assessed for eligibility. A total of 45 key publications, originating from 30 distinct RCTs or major prospective phase II studies, were included in the final qualitative synthesis (Figure 1). Table 1 summarizes the fundamental characteristics of these primary trials and their key ancillary analyses, encompassing landmark TNT trials, immunotherapy-TNT combinations (including SPRING-01 and PRECAM), OP studies, predictive factor analyses (including CINTS-R), de-escalation trials (GRECCAR 4, FOWARC, CONVERT, PROSPECT), and trials evaluating advanced radiotherapy modalities such as proton therapy and MRI-LINAC [11,12,13,21,22,25,26,40,41,42].

#### 3.1.2. Risk of Bias Assessment

Of the 30 included trials, 27 were phase II/III RCTs assessed using RoB 2. All demonstrated low risk of bias in the randomization process and outcome measurement. The three single-arm phase II studies (PRECAM, Averectal, and NORMAL-R) were not formally assessed due to a lack of a comparator. No trial was rated as high risk of bias.

#### 3.1.3. MRI-Based Risk Stratification: Defining Three Clinical Risk Groups

Magnetic resonance imaging is the cornerstone of baseline staging and treatment planning in LARC. The MERCURY and MERCURY II criteria have established that certain MRI features predict higher risks of local recurrence, distant metastasis, and poor response to neoadjuvant therapy. Based on these features, patients can be classified into three risk groups to guide treatment intensity [54].

##### High-Risk Features (Indicate Need for TNT Intensification)

- Threatened or involved mesorectal fascia (MRF+): Tumour or lymph node within ≤1 mm of the mesorectal fascia. Associated with local failure rates > 20% after standard CRT alone.
- cT4 category:
  - T4a: tumour invades the visceral peritoneum (peritoneal reflection). High risk of peritoneal dissemination.
  - T4b: tumour directly invades adjacent organs (bladder, prostate, seminal vesicles, sacrum, vagina, pelvic sidewall). Very high risk of involved margins and local relapse.
- Extramural vascular invasion (EMVI+): Presence of tumour within veins outside the muscularis propria (serpentine appearance, expansion of vessel). Strongly associated with synchronous and metachronous distant metastases.
- Bulky N2 disease: Multiple regional lymph nodes (≥4) or large nodes (>10–15 mm) in the mesorectum. Indicates high tumour burden and risk of residual disease after CRT.
- LPLN+: Nodes in the internal iliac, obturator, or external iliac chains. Size ≥ 7 mm short axis, or ≥5 mm with malignant features (irregular border, mixed signal intensity, loss of fatty hilum). These nodes are not adequately treated by standard TME and may require lateral lymph node dissection (LLND) after TNT.
- Tumour deposits (TD+): Discrete macroscopic tumour nodules within the mesorectum lacking identifiable residual lymph node architecture. They are associated with a higher risk of local recurrence and distant metastases, independent of nodal status. Their presence should be systematically reported on baseline MRI and considered an indication for treatment intensification, including TNT.

##### Intermediate-Risk Features (Standard CRT or TNT May Be Considered)

- cT3 with clear MRF (>1 mm).
- cN1 (1–3 small nodes).
- No EMVI.
- No LPLN enlargement.
- TD−.

##### Low-Risk Features (De-Escalation Possible)

- cT2–3, MRF−, N0–1 (non-bulky), EMVI−, LPLN−.
- Mid-upper rectal location (≥5–10 cm from anal verge).

### 3.2. The Efficacy of Total Neoadjuvant Therapy: A Confirmed Benefit with a Critical Caveat

**Figure 2:**
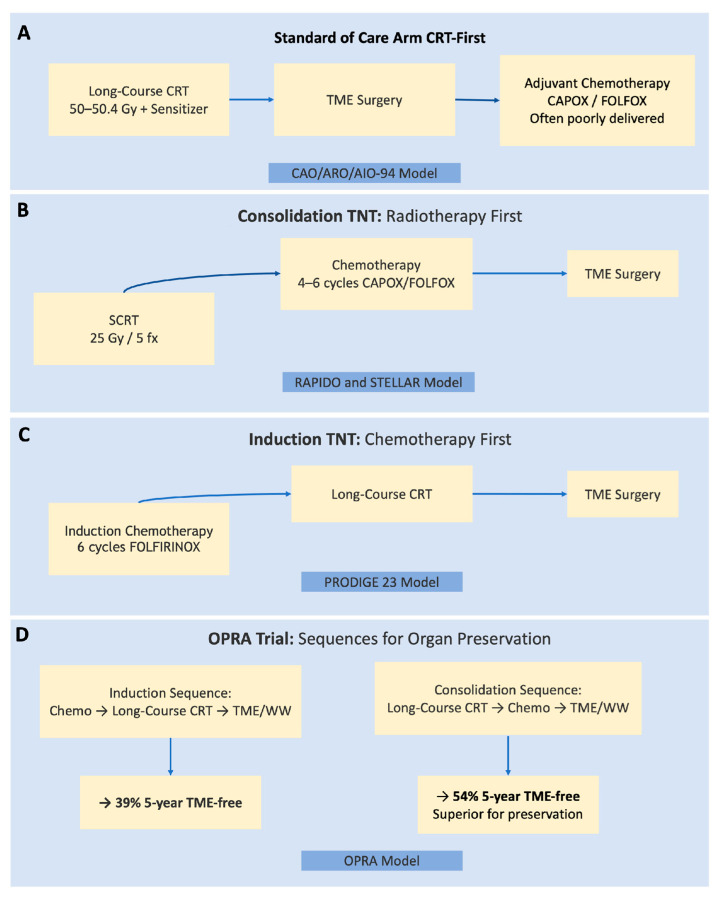
Comparison of standard CRT and TNT sequencing strategies. (**A**) Standard LCRT followed by TME and optional adjuvant chemotherapy. (**B**) Consolidation TNT: short-course radiotherapy (SCRT, 5 × 5 Gy) followed by several cycles of chemotherapy (CAPOX or FOLFOX) and then TME (RAPIDO, STELLAR, TNTCRT-type LCRT consolidation). (**C**) Induction TNT: induction chemotherapy (e.g., FOLFIRINOX in PRODIGE 23) followed by LCRT, then TME and optional adjuvant chemotherapy. (**D**) Organ-preserving TNT with WW: typically consolidation chemotherapy after CRT (OPRA trial), followed by response assessment; patients with CCR enter WW, while those with incomplete response proceed to TME. Abbreviations: CAPOX = capecitabine + oxaliplatin; CCR = clinical complete response; FOLFIRINOX = fluorouracil, leucovorin, irinotecan, oxaliplatin; FOLFOX = fluorouracil, leucovorin, oxaliplatin; LCRT = long-course chemoradiotherapy; SCRT = short-course radiotherapy; TME = total mesorectal excision; TNT = total neoadjuvant therapy; WW = watch-and-wait. Note: Newer trials (TNTCRT, UNION, SPRING-01, PRECAM) follow similar backbone designs with the addition of immunotherapy (PD-1/PD-L1 inhibitors) either concurrently or after SCRT/LCRT.

The efficacy outcomes of the three pivotal phase III trials are summarized in Table 2. As illustrated in Figure 2, these trials evaluated distinct TNT sequences, consolidation (RAPIDO, STELLAR) and induction (PRODIGE 23), all demonstrating that TNT significantly improves disease control compared to standard CRT. Our synthesis confirms that, for high-risk LARC, TNT represents a superior therapeutic strategy compared to traditional CRT followed by surgery and adjuvant chemotherapy (Level of evidence: I).

The PRODIGE 23 trial, utilizing an induction chemotherapy approach (induction FOLFIRINOX → LCRT → TME → mFOLFOX: Figure 2C), demonstrated a significant improvement in 7-year DFS (67.6% vs. 62.5%; HR 0.69) and, critically, a 7-year overall survival (OS) benefit (81.9% vs. 76.1%; HR 0.72), the first OS benefit in the TME era. The pCR rate was more than doubled (27.5% vs. 11.7%) [12,37].

The RAPIDO trial established that a SCRT-based TNT regimen (SCRT → chemotherapy → TME: Figure 2B) significantly reduced 3-year disease-related treatment failure (23.7% vs. 30.4%; HR 0.75), primarily driven by a reduction in distant metastases (20.0% vs. 26.8%) [11]. pCR rates doubled (28% vs. 14%).

The STELLAR trial from an Asian population confirmed the non-inferiority of an SCRT-based TNT approach (SCRT → CAPOX → TME: Figure 2A) compared to LCRT followed by TME and adjuvant chemotherapy, with 3-year DFS rates of 64.5% vs. 62.3%, respectively [13].

The TNTCRT trial provided additional phase III evidence using LCRT-based TNT with CAPOX (one cycle induction, two cycles concurrent, three cycles consolidation). At a median follow-up of 44 months, the 3-year DFS was significantly improved in the TNT arm (77.0% vs. 67.9%; HR 0.623, 95% CI 0.435–0.892, *p* = 0.009). The 3-year metastasis-free survival was also significantly higher (83.0% vs. 74.2%; HR 0.595, *p* = 0.013). The pCR rate was 27.5% in the TNT arm vs. 9.9% in the standard arm (OR 3.436, *p* = 0.0001). Importantly, this trial used LCRT (50–50.4 Gy) rather than SCRT, demonstrating that LCRT-based TNT is also highly effective [14].

These consistent findings across diverse geographical populations and TNT sequencing strategies solidify TNT as the standard of care for high-risk LARC. The primary oncologic benefit is enhanced systemic control, which translates into reduced distant metastases and, as evidenced by PRODIGE 23, an eventual survival advantage. The doubling or tripling of pCR rates is not merely a surrogate but a gateway to organ preservation [34,37].

However, a critical trade-off must be acknowledged and interrogated. The 5-year follow-up of the RAPIDO trial revealed a significantly higher incidence of locoregional recurrences in the experimental TNT arm compared to the standard arm (10% vs. 6%; *p* = 0.027) [38]. This finding has become a central point of debate.

The long-term results of the Polish II trial provide important context. With a median follow-up of 7.0 years, this trial compared SCRT with three cycles of FOLFOX4 versus chemoradiation (50.4 Gy with bolus 5-FU, leucovorin, and oxaliplatin) in cT4 or fixed cT3 rectal cancer. The initial early OS benefit favoring SCRT-based TNT (9% at 3 years) disappeared with longer follow-up; at 8 years, OS was 49% in both groups (HR 0.90; 95% CI 0.70–1.15; *p* = 0.38) [39]. No significant differences were observed in DFS, local failure (35% vs. 32%), or distant metastases (36% vs. 34%). Late complication rates were similar (grade 3+: 11% vs. 9%). These findings suggest that the early OS benefit observed with SCRT-based TNT may not be sustained, and the choice of radiotherapy platform requires careful consideration.

Why did RAPIDO potentially fail on local control? Several hypotheses warrant consideration: (1) Inadequate radiosensitization. The SCRT backbone (5 × 5 Gy) may provide insufficient biological dose intensity for bulky, locally advanced tumors compared to the protracted, radiosensitized course of LCRT. (2) Prolonged interval to surgery. The long interval between radiotherapy and surgery in the consolidation TNT arm may allow for tumor cell repopulation in radioresistant clones. (3) Patient selection. The high-risk population in RAPIDO (e.g., cT4, N2, EMVI+, MRF+) may require the more potent local effects of LCRT; and (4) Loss of adjuvant chemotherapy effect.

This observation has led to nuanced clinical recommendations. ASCO 2024 guidelines issued a conditional preference for LCRT over SCRT within TNT regimens, particularly when maximizing local control is paramount [38]. ESMO 2025 guidelines consider both modalities valid but acknowledge LCRT may be preferred for very high-risk local tumors or when OP is the primary objective.

#### Baseline Heterogeneity Among TNT Trials: A Critical Comparison

A fundamental limitation of cross-trial comparisons of TNT efficacy is the marked heterogeneity in patient populations. Table 3 summarizes the key baseline risk factors for the major phase III TNT trials and selected immunotherapy/de-escalation studies.

As shown in Table 3, the RAPIDO trial enrolled the highest-risk population, with 74% cT4 tumors, 86% cN2, and 53% EMVI+. In contrast, PRODIGE 23 had only 26% cT4 (and a lower proportion of cN2, approximately 70%). STELLAR was intermediate with 37% cT4 and 74% cN2. The TNTCRT trial also enrolled a high-risk population (cT4, cN2, EMVI+, MRF+, or enlarged lateral nodes) and reported a pCR rate of 27.5% with LCRT-based TNT [14].

These differences have profound implications:

I. The absolute pCR rate (28% in RAPIDO vs. 27.5% in PRODIGE 23) appears similar, but the RAPIDO population was much higher risk. Therefore, the relative benefit of TNT over standard CRT may be greater in higher-risk patients.
II. The increased locoregional recurrence seen in RAPIDO (10% vs. 6%) may be partly explained by the high-risk features (cT4, EMVI+), which are known to predispose to local failure, and by the use of SCRT instead of LCRT.
III. De-escalation trials (PROSPECT, CONVERT, FOWARC) intentionally excluded many high-risk patients (e.g., cT4, MRF+), so their results cannot be generalized to the high-risk LARC population.

Consequently, any comparison of outcomes across trials must account for these baseline differences. Clinicians should select TNT regimens based on the patient’s risk profile and the trial that best matches that profile, rather than assuming all TNT strategies are equally effective across all risk strata.

### 3.3. De-Escalation: The PROSPECT Trial and Beyond

Parallel to TNT intensification, the PROSPECT trial established a valid de-escalation option for selected lower-risk patients [40]. In patients with mid-to-upper rectal tumors (cT2 N1, cT3 N0/N1, CRM-negative), neoadjuvant FOLFOX (6 cycles) with selective salvage LCRT for poor response (required in only 9.1%) was non-inferior to standard LCRT for 5-year DFS (80.8% vs. 81.7%). This strategy spares patients the long-term toxicity of pelvic radiation (particularly sexual dysfunction) at the cost of increased acute chemotherapy-related toxicity.

The CONVERT trial further explored radiotherapy omission in LARC with uninvolved mesorectal fascia (MRF−). In this phase III trial of 663 patients, neoadjuvant CAPOX alone (4 cycles) was compared to capecitabine-based nCRT. The non-inferiority of nCT was not formally confirmed due to a very low incidence of local recurrence in both groups (3-year LRRFS: 96.3% for nCT vs. 97.4% for nCRT; HR 1.40, 95% CI 0.53–3.68). However, nCT offered comparable DFS (89.2% vs. 87.9%) and OS (95.0% vs. 94.1%) while significantly reducing grade 2–4 long-term adverse events (16.0% vs. 26.3%, *p* = 0.002) and proctitis (33.6% vs. 41.7%, *p* = 0.049). These findings support radiotherapy omission in carefully selected patients with uninvolved MRF [41].

The FOWARC trial long-term results with a median follow-up of 10 years compared mFOLFOX6 with or without radiation versus fluorouracil plus radiation. The 10-year DFS rates were 52.5%, 62.6%, and 60.5%, respectively (*p* = 0.56), and 10-year LR rates were 10.8%, 8.0%, and 9.6% (*p* = 0.57). Patients achieving pCR had excellent outcomes (10-year DFS 84.3%, OS 92.4%). These long-term data confirm that neoadjuvant mFOLFOX6 alone can be a therapeutic option for LARC without compromising local control or survival [7].

The GRECCAR 4 trial evaluated a response-adaptive strategy: patients with LARC received induction chemotherapy and good responders proceeded directly to surgery without CRT, while poor responders received CRT. This approach demonstrated the feasibility of tailoring treatment intensity based on early response, avoiding unnecessary radiotherapy in good responders. pCR (ypT0) rates varied according to response: 10% in good responders (Arm A, no CRT), 60% in poor responders who received CRT (Arm B), 15% in an intermediate group (Arm C), and 24% overall [42].

These de-escalation and response-adaptive approaches demonstrate that a “one-size-fits-all” approach is obsolete; the availability of both intensification (TNT) and de-escalation (PROSPECT, CONVERT, FOWARC, GRECCAR 4) enables truly personalized treatment.

### 3.4. Immunotherapy in MSS Disease: A Paradigm Shift in Waiting, Not Yet Realized

A transformative, but still investigational, development is the integration of immune checkpoint inhibitors (ICIs) into TNT for MSS rectal cancer. The UNION trial (camrelizumab) [32] and STELLAR II trial (sintilimab) [55] have reported remarkable increases in pCR and CCR rates, approximately doubling them compared to TNT alone (e.g., UNION: 39.8% vs. 15.3%) (Level of evidence: II, pending mature phase III confirmation) [18,20,56]. The Averectal study (avelumab) reported a pCR rate of 36% [47].

The SPRING-01 trial, a randomized phase II trial, compared SCRT followed by sintilimab plus CAPOX versus SCRT followed by CAPOX alone in 98 patients with LARC. The pCR rate was significantly higher in the immunotherapy-containing arm: 59.2% (95% CI 45.4–72.9) vs. 32.7% (95% CI 19.5–45.8) (*p* = 0.015). The complete response rate (pCR + cCR) was also significantly improved (61.2% vs. 32.7%; OR 3.2, 95% CI 1.4–7.5, *p* = 0.0085). Grade 3–4 treatment-related adverse events occurred in 33% vs. 35%, with no treatment-related deaths in the immunotherapy arm. These results are among the highest pCR rates reported in MSS LARC to date [21].

The PRECAM study evaluated short-course nCRT (25Gy/5f) followed by two cycles of CAPEOX and six weekly doses of enzalofilimab (a PD-L1 antibody) in 34 patients with MSS LARC. The pCR rate was 62.5% (20/32), and the major pathologic response rate (TRG 0–1) was 75%. Common adverse events were manageable, with only two grade 3 events (liver function abnormality and thrombocytopenia). These findings further support the potent synergy between short-course radiotherapy and PD-1/PD-L1 blockade [22,55].

The NRG-GI002 trial (long-term results) evaluated pembrolizumab added to TNT in a phase II platform. With longer follow-up, the addition of pembrolizumab was associated with a statistically significant improvement in 3-year OS (95% vs. 87%; HR 0.35, 95% CI 0.12–1.00, *p* = 0.04), but not DFS (64% vs. 64%; HR 0.95, *p* = 0.82). The neoadjuvant rectal (NAR) score improvement was not statistically significant (mean difference 2.9, *p* = 0.21). These results suggest a potential OS benefit that requires confirmation in larger trials [48].

While these results are highly promising and suggest that radiotherapy can act as an in situ vaccine to overcome immune evasion in MSS disease, a cautious interpretation is essential for several reasons: (1) Phase II data predominates: Most of these results are from phase II trials or early analyses of phase III trials with small sample sizes. Mature survival data (DFS, OS) are not yet available for many. (2) Heterogeneous designs: In the UNION trial, the control and experimental arms differed in both systemic therapy and radiotherapy platform. (3) Toxicity signals: The addition of ICIs increases the risk of immune-related adverse events (irAEs). (4) Variability in pCR rates: pCR rates range from 36% (Averectal) to 62.5% (PRECAM), indicating that optimal regimens, sequencing, and patient selection are not yet defined.

Therefore, these results must be considered hypothesis-generating. While they provide a powerful signal and justify rapid further investigation, the addition of immunotherapy to TNT for MSS rectal cancer cannot currently be recommended for routine clinical implementation outside the context of a clinical trial.

### 3.5. Organ Preservation: Proven Efficacy in Expert Centers, Questions of Generalizability

The high pCR rates achieved with TNT have legitimized OP as a viable oncologic strategy. The randomized phase II OPRA trial provided pivotal evidence, demonstrating that a WW approach for good responders yields long-term OP in over 50% of patients without compromising survival [15,43]. Its updated 5-year results are central to clinical practice: (1) TME-free survival: Significantly higher with the consolidation chemotherapy sequence (54% vs. 39% for induction), supporting consolidation as the preferred sequence when OP is a goal; (2) Safety of Salvage: 94% of tumor regrowths occurred within the first 2 years, and salvage TME for regrowth resulted in equivalent 5-year DFS (64%) as immediate TME for incomplete responders; and (3) Predictive response grading: The validation of a three-tier clinical response system (CCR, NCR, ICR) provides a powerful prognostic tool [43]. Patients with a sustained CCR had a 3-year OP rate of 77% and a 3-year DFS of 88%, whereas those with an NCR had rates of 40% and 69%, respectively.

The CAO/ARO/AIO-16 trial provided additional prospective data on OP after TNT, reporting a CCR rate of 36% and a 3-year OP rate of 68% in patients achieving CCR. Patients with sustained CCR had significantly better bowel function (lower LARS/Wexner scores) compared to those undergoing immediate TME [49].

The MONT-R trial also included an ancillary study evaluating transanal endoscopic microsurgery (TEM) versus radical surgery in patients achieving cCR or near-cCR after nCRT. At a median follow-up of 60 months, TEM was associated with significantly faster recovery, better sphincter function (Wexner: 1 vs. 4, *p* = 0.001; LARS: 0 vs. 17, *p* < 0.001), and comparable 5-year DFS (75.6% vs. 80.9%, *p* = 0.658) and OS (93.2% vs. 88.2%, *p* = 0.465). These findings support local excision as an alternative to TME for selected good responders, preserving function without compromising oncologic outcomes [30,31,43].

### 3.6. Predictive Factors and Risk Stratification: Informing Personalization and the Role of ctDNA

Identifying predictors of response is crucial for refining patient selection. A post hoc analysis of the RAPIDO trial identified that the use of TNT itself (OR 2.70), a pretreatment CEA level < 5 µg/L, and a tumor size < 40 mm were independent predictors of achieving a pCR [38]. Achieving pCR was associated with excellent prognosis (5-year OS > 90%).

Conversely, the presence of LPLN+ remains a stubbornly negative prognostic factor, even in the TNT era. A subgroup analysis from the STELLAR trial showed that patients with LPLN+ had inferior 3-year DFS compared to those without (51.7% vs. 66.2%) [28].

The CINTS-R trial represents a major advance in precision medicine for LARC. This multicenter randomized controlled trial uses ctDNA to guide neoadjuvant treatment intensity. In the experimental group, patients with high baseline ctDNA abundance (median VAF ≥ 0.5%) or persistent ctDNA positivity after CRT receive TNT (nCRT plus 4–6 cycles of XELOX), while ctDNA-low-risk patients receive conventional nCRT. Patients with dMMR/MSI-H or TMB-H receive neoadjuvant immunotherapy. The interim analysis of 349 patients demonstrated feasibility and safety: serious adverse events occurred in 10.0% of the experimental group vs. 6.6% of the control group (*p* = 0.316). Notably, 15.7% of TNT-treated patients discontinued chemotherapy due to SAEs, whereas all nCRT recipients completed treatment. Importantly, traditional clinical risk stratification and ctDNA-guided stratification showed substantial discordance: 43.0% of traditionally high-risk patients were classified as ctDNA-low-risk, and 53.1% of traditionally low-risk patients were classified as ctDNA-high-risk. This highlights the potential of ctDNA to refine patient selection beyond clinical factors alone. The primary endpoint (2-year DrTF rate) is awaited [25,26].

### 3.7. Toxicity, Compliance, and Patient-Reported Quality of Life

As summarized in Table 4, the intensified nature of TNT regimens results in a higher incidence of acute grade ≥ 3 toxicities during the neoadjuvant phase, primarily hematological (e.g., neutropenia) and gastrointestinal (e.g., diarrhea), compared to standard LCRT alone. Rates ranged from 26.5% in STELLAR to 47.6% in RAPIDO for the TNT arms [3,11,13,29]. The MONT-R trial reported grade 3–4 toxicity rates of 14.1% with CapeOX vs. 9.3% with capecitabine alone (*p* = 0.095) [28,30,31]. Compliance with the planned preoperative therapy was generally high (>80% completion).

However, comprehensive longitudinal quality of life (QoL) assessments from these trials provide a reassuring and nuanced picture. The RAPIDO QoL study found no significant differences in global health status, bowel function (assessed by the Low Anterior Resection Syndrome score), or late grade ≥ 3 toxicity at 3 years post-surgery between TNT and standard care groups [45]. The PRODIGE 23 analysis revealed that while neoadjuvant FOLFIRINOX transiently reduced global QoL, scores recovered by 2 years and converged with the standard arm [57]. The STELLAR trial reported no clinically significant difference in global QoL or anal function at 6 years [58]. Additionally, a dosimetric comparison from the PRORECT trial suggested proton therapy may significantly reduce the risk of acute GI toxicity [24].

The CONVERT trial demonstrated that nCT alone significantly reduced grade 2–4 long-term AEs (16.0% vs. 26.3%, *p* = 0.002) and proctitis (33.6% vs. 41.7%, *p* = 0.049) compared to nCRT, with comparable DFS and OS [41].

The Polish II trial reported no significant differences in late complication rates between SCRT-based TNT and chemoradiation (grade 3+: 11% vs. 9%, *p* = 0.66) [39].

While TNT imposes a higher acute treatment burden, it does not appear to inflict permanent detriment to long-term QoL for most patients. The decision to pursue TNT involves balancing this transient burden against the long-term benefits of reduced metastasis risk, survival gain, and the chance for organ preservation.

### 3.8. Technological Advances in Radiotherapy: MRI-LINAC and Adaptive Planning

Beyond proton therapy, magnetic resonance-guided radiotherapy (MRI-LINAC) represents a transformative technological leap. Chen et al. developed an efficient library of reference plans (LoRP) strategy for MRI-guided adaptive radiotherapy of rectal cancer [59]. This approach involves preparing multiple reference plans based on diverse bladder shapes; for each fraction, a plan is selected based on daily bladder filling. Compared to fully adaptive (adapt-to-shape) strategies, LoRP reduced treatment session duration by more than a third (>20 min) while maintaining acceptable target coverage (94% vs. 95% for fully adaptive, and 92% of LoRP plans achieved acceptable dose criteria vs. 74% for conventional couch shift). This strategy enhances treatment efficiency, patient comfort, and enables real-time adaptation to anatomical changes.

### 3.9. Synthesis of Key Findings

**Figure 3:**
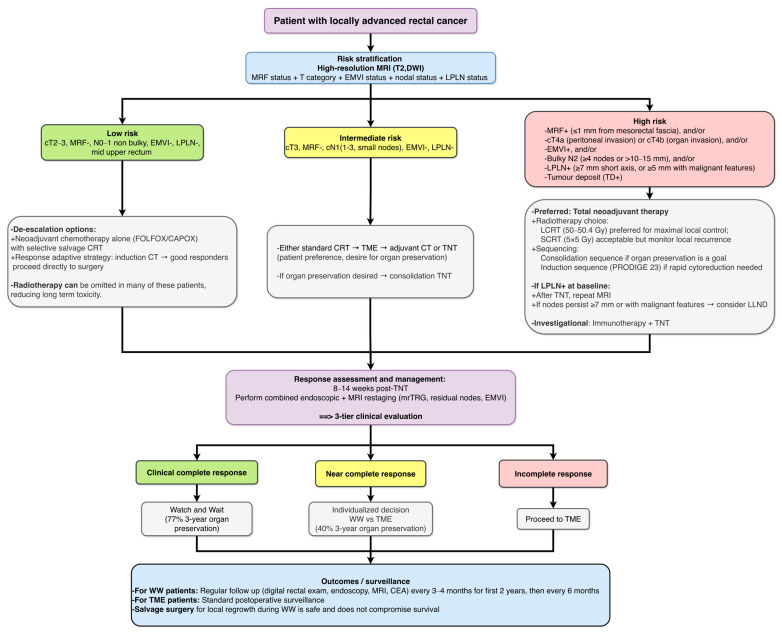
MRI-based risk-adapted treatment algorithm for locally advanced rectal cancer. This algorithm integrates baseline pelvic MRI features (MRF status, cT category, EMVI, nodal burden, LPLN size/morphology) to classify patients into high-, intermediate-, or low-risk groups. Treatment intensity is then tailored accordingly: (1) High-risk LARC (MRF+, cT4, EMVI+, bulky N2, LPLN+, or TD+): TNT is preferred. LCRT-based TNT is favoured for maximal local control (PRODIGE 23, TNTCRT); SCRT-based TNT (RAPIDO, STELLAR) is an option but carries a higher risk of locoregional recurrence. Consolidation chemotherapy is preferred when organ preservation is a goal (OPRA). For persistent LPLN+ after TNT, LLND should be considered (Ogura criteria). Immunotherapy TNT combinations (UNION, SPRING 01, PRECAM) remain investigational (dashed lines). (2) Intermediate-risk LARC (cT3, MRF−, cN1, EMVI−, LPLN−, and TD−): Standard CRT or TNT are both acceptable. TNT with consolidation may be offered to patients who strongly desire organ preservation. (3) Low-risk LARC (cT2–3, MRF−, N0–1 non bulky, EMVI−, mid upper rectum): De-escalation options include neoadjuvant chemotherapy alone (PROSPECT, CONVERT, FOWARC) with selective salvage CRT, or response adaptive strategies (GRECCAR 4). Radiotherapy omission reduces long term toxicity without compromising survival. Post TNT response assessment (8–14 weeks) uses a three-tier clinical system (CCR, NCR, ICR) combined with MRI (mrTRG, nodal status, EMVI clearance): (1) CCR → WW or local excision (TEM, MONT R trial); (2) NCR → WW with intensive surveillance or TME after shared decision; or (3) ICR → prompt TME. Abbreviations: CCR = clinical complete response; CRT = chemoradiotherapy; CT = chemotherapy; EMVI = extramural vascular invasion; ICR = incomplete response; LARC = locally advanced rectal cancer; LCRT = long course chemoradiotherapy; LLND = lateral lymph node dissection; LPLN = lateral pelvic lymph node; MRF = mesorectal fascia; MRI = magnetic resonance imaging; NCR = near complete response; SCRT = short course radiotherapy; TEM = transanal endoscopic microsurgery; TME = total mesorectal excision; TNT = total neoadjuvant therapy; WW = watch and wait.

The integrated analysis of the included trials yields several key conclusions that collectively inform the current management paradigm for locally advanced rectal cancer (Figure 3).

First, total neoadjuvant therapy improves systemic control for high-risk LARC. The RAPIDO and PRODIGE 23 trials provide Level I evidence that TNT reduces distant metastases and improves disease-related treatment failure or DFS compared to standard CRT, with PRODIGE 23 now demonstrating an OS benefit [11,12,37]. The STELLAR trial supports the non-inferiority of an SCRT-based TNT regimen for 3-year DFS compared to LCRT-based therapy [13]. The TNTCRT trial provides additional phase III evidence for LCRT-based TNT with CAPOX [14].

Second, TNT carries an inherent trade-off between systemic and local control. While improving distant metastasis rates, some TNT regimens, particularly the SCRT-based consolidation approach used in RAPIDO, are associated with a higher risk of locoregional recurrence compared to standard chemoradiotherapy. The Polish II trial showed that early OS benefits of SCRT-based TNT were not sustained with longer follow-up, with 8-year OS of 49% in both arms [39].

Third, immunotherapy shows highly promising activity in MSS disease, but these results remain investigational. The UNION, STELLAR II, SPRING-01, and PRECAM trials demonstrate that adding a PD-1/PD-L1 inhibitor to SCRT-based TNT markedly increases pCR and cCR rates (approximately 40–60% vs. 15–25%) in MSS rectal cancer [18,20]. SPRING-01 reported the highest pCR rate (59.2%) in a randomized trial to date. However, these findings are primarily from phase II or early-phase III studies with heterogeneous designs and without mature survival data.

Fourth, organ preservation is confirmed as a safe and viable oncologic strategy in expert centers. Prospective trials including OPRA, OPERA, and CAO/ARO/AIO-16 demonstrate that non-operative management for good responders yields long-term OP in over 50% of selected patients without compromising survival [15,16,49]. The MONT-R TEM study further supports local excision as an alternative to TME for good responders [28,30,31].

Fifth, therapeutic sequence significantly impacts OP rates. The consolidation chemotherapy sequence yields higher rates of both pCR and OP compared to the induction sequence [15,46].

Sixth, short-course radiotherapy has been validated as an efficient TNT backbone, but its use requires careful consideration of local-control trade-offs. The STELLAR and Polish II trials confirm that SCRT followed by chemotherapy is non-inferior to conventional long-course radiotherapy for long-term survival, offering a shorter and more resource-efficient treatment platform [13,35,45,60]. However, the Polish II long-term results showed no sustained OS benefit.

Seventh, predictive factors and refined response grading significantly advance personalization. The CINTS-R trial is pioneering ctDNA-guided risk stratification, with interim analysis confirming feasibility and safety [25,26]. The OPRA three-tier clinical response system provides a powerful prognostic tool [20,43].

Eighth, advanced radiotherapy modalities can mitigate treatment toxicity. Dosimetric data from the PRORECT trial suggest that proton therapy could significantly reduce acute gastrointestinal toxicity [23]. MRI-LINAC with library of reference plans enables efficient adaptive radiotherapy, reducing treatment session duration while maintaining target coverage [46].

Ninth, de-escalation and response-adaptive strategies are feasible in selected patients. The PROSPECT, CONVERT, FOWARC, and GRECCAR 4 trials demonstrate that radiotherapy can be safely omitted or adapted based on response in lower-risk LARC, reducing long-term toxicity without compromising survival.

Tenth, specific clinical subgroups demand tailored approaches. LPLN+ remains a negative prognostic factor despite TNT, warranting consideration of treatment intensification [45]. Elderly patients require individualized care focused on function preservation, often leveraging the efficacy of radiotherapy and organ preservation strategies guided by Comprehensive Geriatric Assessment [9,27].

Eleventh, real-world evidence supports feasibility and tolerability. Large cohort studies, such as the Swedish nationwide LARCT-US project, confirm that SCRT-based TNT (with a modified, shorter chemotherapy course) is feasible, effective, and associated with manageable toxicity in routine clinical practice [17].

Finally, long-term QoL is preserved with TNT, as despite higher acute toxicity, it does not impair long-term global quality of life, bowel, or anal function compared to standard CRT [45,57,58].

## 4. Discussion

### 4.1. Implementation of Total Neoadjuvant Therapy

For patients with high-risk LARC, TNT is now a preferred strategy supported by landmark phase III trials [11,12,13]. By delivering all systemic chemotherapy preoperatively, TNT targets micrometastatic disease earlier, improves treatment compliance, and achieves superior tumor downstaging, tripling pCR rates and creating opportunities for organ preservation [11,12,13,46].

However, the critical trade-off revealed by the RAPIDO trial, improved systemic control at the cost of increased locoregional recurrence (10% vs. 6%), must centrally inform treatment decisions [11,38]. The Polish II trial long-term results further caution that early OS benefits may not be sustained. This observation reframes TNT not as a monolithic regimen but as a flexible framework requiring deliberate optimization of two sequential choices.

The first decision is the radiotherapy platform. The STELLAR trial validated SCRT-based TNT as non-inferior to LCRT for 3-year DFS, offering efficiency and resource savings [13]. Real-world data from the LARCT-US cohort further support its feasibility [17]. However, in light of RAPIDO’s local failure signal and the Polish II long-term results, LCRT may be preferred for tumors at the highest risk of local recurrence (e.g., low-lying tumors, MRF+), where protracted radiosensitization may be advantageous. The TNTCRT trial provides strong evidence for LCRT-based TNT with CAPOX, demonstrating significant DFS and MFS benefits [14].

The second decision is treatment sequencing. Induction chemotherapy (PRODIGE 23) facilitates rapid cytoreduction for symptomatic or bulky tumors [12]. Consolidation chemotherapy (RAPIDO, OPRA) maximizes the likelihood of deep clinical response and is therefore preferred when OP is the primary [11,15].

The optimal chemotherapy backbone also requires consideration. The MONT-R trial demonstrated that adding oxaliplatin to capecitabine during nCRT improves tumor regression but does not translate into DFS or OS benefits, suggesting that the intensification of local therapy does not always improve systemic outcomes [30,31].

Thus, TNT application requires individualization: selecting the appropriate radiotherapy backbone based on local risk, then the optimal sequence based on treatment objectives, always balancing the systemic-local control trade-off (Figure 3).

Crucially, the marked heterogeneity in baseline risk factors across TNT trials must be acknowledged when interpreting outcomes (Table 3). RAPIDO enrolled a much higher-risk population (74% cT4, 53% EMVI+) than PRODIGE 23 (26% cT4). Despite this, both trials achieved similar pCR rates (approximately 28%). This suggests that the relative benefit of TNT over standard CRT may be greater in higher-risk patients, but also that the trade-off with locoregional control is more pronounced in such patients. Therefore, a regimen that works well in a lower-risk population (e.g., PRODIGE 23) cannot be assumed to have the same safety profile in a very high-risk population. Clinicians should match the TNT regimen to the patient’s risk profile, considering both the radiotherapy platform (SCRT vs. LCRT) and the intensity of chemotherapy.

### 4.2. Organ Preservation Paradigm and MRI-Guided Selection

#### 4.2.1. Organ Preservation

Organ preservation has become a standard option within specialized multidisciplinary care, redefining success to include functional integrity without compromising oncologic outcomes for selected patients.

The three-tier response grading system validated by the OPRA trial (CCR/NCR/ICR) is the cornerstone of precision patient selection [43]. This framework moves beyond the binary “CCR versus not” to enable risk-adapted management: (1) Sustained CCR: WW can be offered with high confidence; (2) NCR: WW remains feasible but requires intensive surveillance; and (3) ICR: Prompt TME is indicated.

The MONT-R TEM study provides strong evidence that local excision (transanal endoscopic microsurgery) is a safe alternative to TME for selected good responders, offering better functional outcomes (Wexner: 1 vs. 4, LARS: 0 vs. 17) with comparable 5-year DFS (75.6% vs. 80.9%) and OS (93.2% vs. 88.2%) [30,31].

Technical innovations further expand OP boundaries. The OPERA trial demonstrated that a CXB boost after CRT significantly improves OP rates (81% vs. 66% at 3 years) for small, distal tumors [16].

A critical caveat regarding generalizability must be emphasized. The outstanding outcomes from OPRA and OPERA were achieved in highly selected patients managed within high-volume expert centers. These results may not be immediately generalizable to all clinical settings.

#### 4.2.2. Integrating MRI into Clinical Decision-Making

The use of high-quality baseline pelvic MRI is mandatory for all patients with LARC. Beyond simple TNM staging, MRI identifies key biological risk factors that dictate treatment intensity: (1) MRF+ predicts local failure even after good systemic therapy; such patients require TNT with careful attention to the radiotherapy platform (LCRT may be preferred over SCRT); (2) EMVI+ is a powerful predictor of distant metastasis; these patients derive the greatest benefit from the intensified systemic therapy of TNT (either induction or consolidation); (3) LPLN+ cannot be managed by standard TME alone. After TNT, post-treatment MRI should reassess lateral nodes: if they persist ≥ 7 mm or show malignant features, LLND should be considered (Ogura criteria); and (4) cT4b with organ invasion requires TNT and a multidisciplinary surgical plan (en-bloc resection may be necessary).

The three-risk-group framework (Table 2) allows clinicians to move beyond a one-size-fits-all approach. For high-risk patients, TNT is the preferred strategy, and participation in clinical trials of immunotherapy-TNT combinations is encouraged. For intermediate-risk patients, either standard CRT or TNT is acceptable, with the choice influenced by the patient’s desire for organ preservation (consolidation TNT preferred). For low-risk patients, de-escalation strategies (chemotherapy alone, selective CRT, response-adaptive care) are safe and reduce long-term toxicity.

MRI is also essential for post-TNT response assessment (mrTRG, residual nodal status, EMVI clearance). The combination of endoscopic (CCR/NCR/ICR) and MRI findings provides the most accurate prediction of pathological complete response and guides the watch-and-wait or local excision decision.

### 4.3. Immunotherapy in MSS Disease: Investigational Only

The integration of PD-1 inhibitors with TNT for MSS rectal cancer represents a highly promising but still investigational strategy [18,20]. The SPRING-01 trial reported a pCR rate of 59.2% with sintilimab-containing TNT, and the PRECAM study reported a pCR rate of 62.5% with enzalofilimab. These are among the highest pCR rates ever reported in MSS LARC [21,22].

However, a rigorously cautious interpretation is warranted for several reasons: (1) Maturity of Data: These results are predominantly from phase II trials. Mature survival data (DFS, OS) are not yet available. (2) Heterogeneous Designs: Different radiotherapy platforms (SCRT vs. LCRT), chemotherapy backbones, ICI agents, and sequencing are being used. (3) Toxicity Considerations: The addition of ICIs introduces irAEs. (4) Cost and Access: These regimens are substantially more expensive. (5) Variability in pCR rates (36–62.5%) indicates that the optimal regimen is not yet defined.

Therefore, while these results are exceptionally promising, the addition of immunotherapy to TNT for MSS rectal cancer must currently be considered investigational outside of a clinical trial context.

### 4.4. Predictive Biomarkers and ctDNA-Guided Therapy: CINTS-R

Accurate response assessment is the cornerstone of organ preservation. While the OPRA trial’s three-tier clinical response grading system represents a major advancement, the inherent challenge persists that a CCR does not always equate to a pCR [43].

The CINTS-R trial represents a paradigm shift toward ctDNA-guided precision neoadjuvant therapy. By using baseline ctDNA abundance and post-CRT ctDNA clearance to stratify patients into TNT versus conventional nCRT (or immunotherapy for dMMR/TMB-H), this trial addresses the critical need for dynamic, biology-driven treatment adaptation. The interim analysis confirms feasibility and safety, and the final results (2-year DrTF rate) are eagerly awaited. If positive, ctDNA-guided therapy could become the new standard for personalizing neoadjuvant treatment intensity [25,26].

The Immunoscore biopsy, which quantifies immune cell densities within the tumor microenvironment, has also been validated as a predictor of local regrowth risk [61].

These sophisticated tools must be integrated with established clinicopathological factors, including pre-treatment CEA < 5 µg/L and tumor size < 40 mm [38].

### 4.5. Technological Advances in Radiotherapy: MRI-LINAC

MRI-guided radiotherapy (MRI-LINAC) represents a transformative technological leap. The library of reference plans (LoRP) strategy developed by Chen et al. addresses a key limitation of MRI-LINAC, the time required for full adaptive replanning. By preparing multiple reference plans based on varying bladder shapes and selecting the most appropriate plan for daily anatomy, LoRP reduced treatment session duration by >20 min compared to fully adaptive strategies, while maintaining acceptable target coverage (94% vs. 95%). This enhances patient comfort, reduces intrafraction motion, and improves the feasibility of daily adaptation in routine clinical practice [46].

### 4.6. Management of Specific Subgroups: LPLN+ and Elderly

Two persistent and complex clinical scenarios demand focused, nuanced strategies.

First, although LPLN+ is often considered a surgical rather than a TNT topic, it directly impacts both TNT decision-making and the feasibility of organ preservation. LPLN+ disease persists as a significant negative prognostic factor even after potent TNT, as evidenced by subgroup analyses from STELLAR [45]. The presence of LPLN+ may indicate the need for intensified TNT and, if nodes persist after neoadjuvant therapy, LLND becomes necessary, which precludes a watch-and-wait strategy. Therefore, the role and ideal timing of LLND in patients who have received TNT must be highly individualized, guided by meticulous multidisciplinary review of post-treatment MRI.

Second, the management of elderly and potentially frail patients requires a paradigm distinct from chronological age alone. A Comprehensive Geriatric Assessment is mandatory to objectively distinguish fit from frail individuals [9,27]. For fit older adults, curative-intent TNT remains appropriate. For the frail, the primary therapeutic aim shifts towards optimizing function and quality of life, with strategies including CRT with OP intent, definitive radiotherapy alone, or CRT followed by local excision.

### 4.7. Health Economics and Real-World Feasibility

The adoption of TNT, particularly with novel agents and advanced technologies, raises significant questions of sustainability and equity. The cumulative cost of intensified chemotherapy, ICIs (when used), CXB boosts, and intensive surveillance protocols for WW is substantial. Rigorous cost-effectiveness analyses are urgently needed to determine the value of these strategies across different healthcare systems. In low- and middle-income countries, the routine implementation of such resource-intensive protocols is currently unfeasible. Therefore, future research and guideline development must consider not only efficacy but also affordability and accessibility, ensuring that advances in rectal cancer care do not exacerbate global health inequities. Rigorous cost-effectiveness analyses are urgently needed.

### 4.8. Limitations, Clinical Implications and Future Directions

#### 4.8.1. Review Limitations

This review has several limitations. First, due to significant clinical and methodological heterogeneity across studies (chemotherapy regimens, radiotherapy schedules, sequencing strategies, and patient populations), a formal quantitative meta-analysis was not performed. Second, publication bias was not formally assessed, although we attempted to minimize this by searching conference abstracts (ASCO/ESMO 2020–2025). Third, certainty of evidence according to the GRADE framework was not evaluated, as this review was designed as a narrative synthesis rather than a guideline-driven meta-analysis. Fourth, the three-tier clinical response system (CCR/NCR/ICR) has been validated only in the OPRA trial and requires external validation. Fifth, most immunotherapy-TNT results derive from phase II trials with small sample sizes and no mature survival data, limiting their generalizability. Sixth, the generalizability of organ preservation outcomes from expert centers (OPRA, OPERA) to community practice remains unproven. Finally, the CINTS-R trial results are interim only; final conclusions await 2-year DrTF data.

#### 4.8.2. Clinical Implications and Future Directions

Based on this comprehensive review, managing LARC should now be guided by evidence-based, yet nuanced, recommendations (Figure 3). It must be emphasized upfront that surgery, specifically TME, remains the durable and sustainable curative treatment for LARC. The following recommendations are intended to optimise outcomes within a surgical framework, recognising that most landmark TNT trials were led by surgical investigators.

For patients with high-risk LARC (cT4, cN2, EMVI+, MRF+): TNT is a preferred approach to maximize systemic control [11,12,13]. However, TNT does not replace the need for TME in the majority of patients; rather, it improves downstaging and reduces distant metastases. The choice between SCRT and LCRT within TNT should balance systemic benefits against local control. LCRT may be favored for tumors at the highest risk of local failure (e.g., low-lying tumours, MRF+). LCRT-based TNT with CAPOX (as in TNTCRT) is a valid and effective option [14].

When OP is a treatment goal: The consolidation sequence is preferred [13,15]. Response should be evaluated 8–14 weeks after completing TNT using a structured three-tier clinical response system (CCR, NCR, ICR) [20,43]. A WW approach can be offered to patients who achieve a sustained CCR and can be cautiously considered for selected patients with NCR, only within a strict, protocolized surveillance pathway in a specialized multidisciplinary center [15,43,57]. Local excision is a safe alternative to TME for selected good responders (MONT-R TEM study) [30,31]. Crucially, organ preservation is reserved for a minority of highly selected patients; for all others, prompt TME remains the standard of care.

Immunotherapy in MSS disease (investigational only): Adding a PD-1/PD-L1 inhibitor to TNT appears promising for increasing CCR rates in MSS patients [18,20]; however, this approach should currently be considered investigational and limited to clinical trials. The high pCR rates reported in SPRING-01 and PRECAM are encouraging but require phase III confirmation [21,22].

For lower-risk LARC (e.g., MRF−, cT3N+ without high-risk features): De-escalation strategies including radiotherapy omission (PROSPECT, CONVERT, FOWARC, OCUM) or response-adaptive approaches (GRECCAR 4) are valid options that reduce long-term toxicity. The OCUM trial prospectively validated that patients with a safe distance (>1 mm) between the tumor or suspicious nodes and the mesorectal fascia (mrMRF−) can undergo upfront TME without neoadjuvant chemoradiotherapy, achieving a 5-year locoregional recurrence rate of only 2.9% [40,41,42,50,53].

ctDNA-guided therapy (investigational): ctDNA-guided therapy (CINTS-R) represents a promising future direction for personalizing neoadjuvant treatment intensity, but remains investigational pending final results.

Vulnerable populations: For elderly patients, a CGA is essential [27]. For patients with LPLN+, treatment should involve intensified TNT with personalized decisions regarding lateral lymph node dissection [45].

#### 4.8.3. Future Research Priorities

The path forward in research and clinical practice will continue to evolve, emphasizing not only improved outcomes but also broader access and equity in care, and will prioritize several key areas:

(1) Immunotherapy confirmation: Phase III confirmatory trials with long-term survival data are urgently needed before immunotherapy-TNT combinations can be adopted as standard of care [18,20].
(2) ctDNA-guided therapy: The final results of the CINTS-R trial (2-year DrTF rate) will determine whether ctDNA-guided risk stratification should become standard practice.
(3) Biomarker integration: Validation and clinical implementation of Immunoscore, ctDNA dynamics, and radiomic signatures are paramount.
(4) Understanding and mitigating trade-offs: Further research is required to elucidate the mechanisms behind increased locoregional recurrence risk and optimize radiotherapy techniques.
(5) Health economic analysis: Rigorous cost-effectiveness analyses of TNT, OP strategies, and novel technologies are essential.
(6) Trials for specific subgroups: Dedicated prospective trials are required for elderly/frail patients and those with LPLN+.
(7) Radiotherapy advancement: Continued optimization of techniques, including proton therapy [23], MRI-guided adaptive radiotherapy, and CXB boosts [22], is crucial [16,23].

## 5. Conclusions

Surgery, specifically TME, remains the durable and sustainable curative treatment for LARC. The majority of landmark trials that established TNT were led by surgical investigators, reflecting the central role of surgery in achieving a cure. Nevertheless, the management of LARC has entered an era of remarkable sophistication, driven by conclusive evidence that TNT improves systemic control and, as shown by PRODIGE 23 and TNTCRT, provides DFS and OS benefits for high-risk disease. For carefully selected patients who achieve a clinical complete response after TNT, W&W or TEM offers equivalent oncologic outcomes with superior functional preservation, as demonstrated by the OPRA, OPERA, MONT-R, and CAO/ARO/AIO-16 trials. Organ preservation is a validated and durable option in expert centers, but it does not replace the fundamental role of surgery for the vast majority of patients.

However, this review has deliberately highlighted the nuances and trade-offs that accompany this progress. The increased locoregional recurrence risk in the RAPIDO trial and the lack of sustained OS benefit in the Polish II trial serve as critical counterbalances. The dramatic pCR rates from immunotherapy-TNT combinations (SPRING-01: 59.2%; PRECAM: 62.5%), while paradigm-shifting in potential, remain investigational and require rigorous confirmatory trials. The MONT-R trial reminds us that adding oxaliplatin to nCRT improves tumor regression but not survival, highlighting the need to distinguish between surrogate endpoints and true clinical benefit.

The CINTS-R trial represents the future of precision medicine in LARC, using ctDNA to dynamically guide treatment intensity. The final results of this trial will be pivotal in determining whether biomarker-driven personalization can improve outcomes and reduce overtreatment.

We now possess more potent therapeutic tools and more refined lenses for assessment. The future of LARC care lies in the seamless synthesis of these elements: selecting the most effective yet best-tolerated strategy based on dynamic biomarkers (ctDNA, Immunoscore), advanced imaging (MRI, MRI-LINAC), and comprehensive patient assessment, with the unwavering dual objectives of achieving cure and preserving quality of life. This review charts the evidence-based, yet cautious, path toward that future of precision oncology, acknowledging both the remarkable progress made and the critical questions that remain, always with the understanding that surgery remains the cornerstone of curative therapy.

“The future of rectal cancer management no longer lies in choosing between surgery and non-surgery, but in dynamically integrating systemic therapy, radiotherapy, biomarkers, and response assessment to deliver cure with maximal functional preservation.”

## Figures and Tables

**Table 1:** Characteristics of included randomized trials and key studies.

Trial/Analysis (Primary Publication)	Phase	Sample Size	Control Arm	Experimental Arm	Primary Endpoint(s)
Historical Standard Treatment					
CAO/ARO/AIO-94 [4]	III	823	Postop CRT	Preop CRT	OS
EORTC 22921 [5]	III	1011	Preop RT alone	Preop CRT → ±Adj CT	OS, DFS
Stockholm III [6]	III	550	SCRT (5 × 5 Gy) with immediate surgery	SCRT with delayed surgery (4–8 weeks)	Local recurrence, survival
CAO/ARO/AIO-04 [7]	III	1265	5-FU + LCRT	5-FU + oxaliplatin + LCRT	3-year DFS
Landmark Phase III TNT Trials					
RAPIDO [11,38]	III	920	LCRT → TME (±Adj CT)	SCRT → CT (CAPOX/FOLFOX) → TME	3-year DrTF
PRODIGE 23 [12,37]	III	461	LCRT → TME → Adj CT	6× FOLFIRINOX → LCRT → TME → 6× mFOLFOX6	3-year DFS
STELLAR [13]	III	599	LCRT → TME → 6× CAPOX	SCRT → 4× CAPOX → TME → 2× CAPOX	3-year DFS
TNTCRT [14]	III	458	LCRT + capecitabine → TME → Adj CAPOX	LCRT + 6× CAPOX (1 ind, 2 conc, 3 cons) → TME	3-year DFS
Polish II long-term [39]	III	515	LCRT + oxaliplatin	SCRT + 3× FOLFOX4 → TME	OS, DFS, local failure
Neoadjuvant Chemotherapy Without Routine RT (De-escalation)					
PROSPECT [40]	III	1194	LCRT (5.5 weeks) → TME	mFOLFOX6 (6 cycles) → TME (± selective LCRT)	5-year DFS (NI)
CONVERT [41]	III	663	LCRT + capecitabine → TME	4× CAPOX → TME	3-year LRRFS (NI)
GRECCAR 4 [42]	II	148	Response-adaptive (induction CT → good responders → surgery; poor responders → CRT)	-	pCR, OP rate
Immunotherapy-TNT Trials					
UNION [18]	III	231	LCRT → 2× CAPOX → TME → 6× CAPOX	SCRT → 2× CAPOX + Camrelizumab → TME → 6× CAPOX + Camrelizumab	pCR
STELLAR II [20]	II	218	SCRT → 4× CAPOX → TME/WW → 2× CAPOX	SCRT → 4× CAPOX + Sintilimab → TME/WW → 2× CAPOX + Sintilimab	CR rate
SPRING-01 [21]	II	98	SCRT → 6× CAPOX → TME	SCRT → 6× CAPOX + Sintilimab → TME	pCR
PRECAM [22]	II	34	(single-arm)	SCRT → 2× CAPEOX + 6× Enzalofilimab → TME	pCR
Organ Preservation and Sequencing					
OPRA [15]	II	324	Induction: CRT → TME/WW	Consolidation: CRT → TME/WW	3-year DFS; OP
OPERA [16]	III	148	LCRT + EBRT Boost → TME/LE/WW	LCRT + CXB Boost → TME/LE/WW	3-year OP rate
MONT-R TEM [30,31]	Prospective case–control	80	Radical surgery (TME)	TEM local excision (cCR/near-cCR)	5-year DFS
Predictive Factor and Subgroup Analyses					
OPRA Response Grade [43,44]	Post hoc	324	Analysis correlating 3-tier response with outcomes	-	OP and DFS by CCR/NCR/ICR
STELLAR LPLN Analysis [28]	Post hoc	599	Subgroup analysis of LPLN+ patients	-	Outcomes in LPLN+
RAPIDO pCR Analysis [45]	Post hoc	920	Analysis of factors associated with pCR	-	Factors for pCR; prognosis of pCR
CINTS-R [25,26]	III RCT	470 (planned)	Conventional nCRT	ctDNA-guided: high-risk → TNT; low-risk → nCRT; dMMR/TMB-H → immunotherapy	2-year DrTF
Radiotherapy Modality					
PRORECT [23]	Dosimetric	128 (plan)	Photon CRT	Proton CRT	Dosimetric comparison; predicted acute ≥ G2 GI toxicity
MRI-LINAC LoRP [46]	Retrospective	10 pts (50 fractions)	Conventional couch shift/fully adaptive	Library of reference plans (LoRP)	Target coverage, treatment time
Other Key Trials					
Averectal [19,47]	II	44	Single-arm: SCRT → 6× mFOLFOX-6 + Avelumab → TME	-	pCR vs. historical
NRG-GI002 [48]	II platform	178 (EA1), 185 (EA2)	FOLFOX → CRT → TME	EA2: + Pembrolizumab during CRT	NAR score reduction
CAO/ARO/AIO-16 [49]	II	91	Single-arm: CRT → 3× FOLFOX → Response → TME/WW	-	CCR rate
CAO/ARO/AIO-12 [46]	II	306	Induction FOLFOX → CRT → TME	CRT → Consolidation FOLFOX → TME	pCR rate
FOWARC 10-year [50]	III	495	5-FU + LCRT	mFOLFOX6 ± LCRT	10-year DFS
TORCH [51]	II	130 (planned)	ICI before vs. after SCRT	ICI after SCRT	CR
NORMAL-R [33]	II	19	SCRT → CT → non-operative for CCR	-	cCR at 1 year
NOMINATE [52]	II	66 (planned)	CRT → 6× CapeOx	3× CapeOx + Bev → CRT → 3× CapeOx	pCR/CCR ≥ 2 years
OCUM [53]	Prospective	1099	Upfront TME (mrMRF−, low-risk)	nCRT → TME (mrMRF+ and/or cT4 and/or lower third cT3, high-risk)	5-year LR rate

CAPEOX = Capecitabine + Oxaliplatin; conc = concurrent; cons = consolidation; ind = induction; LoRP = Library of Reference Plans; LRRFS = locoregional recurrence-free survival; NAR = Neoadjuvant Rectal score; NI = non-inferiority; TEM = transanal endoscopic microsurgery.

**Table 2:** Efficacy of key treatment strategies.

Trial/Analysis	Primary Efficacy Endpoint	pCR/cCR Rate	Key Efficacy Insights
RAPIDO [11]	3-year DrTF: 23.7% vs. 30.4% *	28% vs. 14% *	TNT reduced distant metastases (20.0% vs. 26.8%) and improved DrTF.
PRODIGE 23 [12]	7-year DFS: 67.6% vs. 62.5%; 7-year OS: 81.9% vs. 76.1%	28% vs. 12% *	TNT improved DFS, reduced distant metastases (17.8% vs. 25.5%), and showed OS benefit.
STELLAR [28]	3-year DFS: 64.5% vs. 62.3% (NI)	21.8% vs. 12.3% *	SCRT-based TNT was non-inferior to LCRT-based therapy.
TNTCRT [14]	3-year DFS: 77.0% vs. 67.9% * (HR 0.623); 3-year MFS: 83.0% vs. 74.2% *	27.5% vs. 9.9% *	LCRT-based TNT with CAPOX significantly improved DFS, MFS, and pCR.
PROSPECT [40]	5-year DFS: 80.8% vs. 81.7% (NI)	21.4% vs. 24.0% (NS)	FOLFOX with selective LCRT non-inferior to routine LCRT; 9.1% required salvage LCRT.
CONVERT [41]	3-year LRRFS: 96.3% vs. 97.4% (NI not formally met)	~20% vs. ~22%	nCT offered comparable DFS/OS with significantly less long-term toxicity.
FOWARC 10-year [50]	10-year DFS: 60.5–62.6% vs. 52.5% (NS); 10-year LR: 9.6% vs. 10.8% (NS)	~28% vs. ~14% *	mFOLFOX6 alone non-inferior to CRT at 10 years; pCR predicts excellent survival.
GRECCAR 4 [42]	5-year DFS (response-adaptive)	ypT0 rates: Arm A: 10%, Arm B: 60%, Arm C: 15%, overall: 24%	Good responders to induction CT can avoid CRT without compromising outcomes.
UNION [18]	-	39.8% vs. 15.3% *	Adding camrelizumab to SCRT + CAPOX nearly tripled pCR rate in MSS.
STELLAR II [20]	-	CR: 44.0% vs. 22.9% *	Adding sintilimab increased cCR rate in MSS.
SPRING-01 [21]	-	pCR: 59.2% vs. 32.7%; CR: 61.2% vs. 32.7%	Adding sintilimab to SCRT + CAPOX significantly increased pCR and CR rates (*p* = 0.015).
PRECAM [22]	-	pCR: 62.5%; MPR (TRG 0–1): 75%	Short-course nCRT + enzalofilimab achieved highest pCR in MSS LARC to date.
NRG-GI002 [48]	NAR score diff: 2.9 (*p* = 0.21); 3-year OS: 95% vs. 87% *	Not reported	Pembrolizumab added to TNT improved 3-year OS but not DFS; NAR reduction not significant.
Averectal [19,47]	-	pCR: 36%	SCRT + mFOLFOX-6 + avelumab achieved 36% pCR.
OPRA [15]	5-year DFS: ~70% (both arms)	CCR/NCR: 76% vs. 72%	5-year TME-free survival: 54% (Consolidation) vs. 39% (Induction) *; Consolidation favored for OP.
OPERA [16]	-	CCR/NCR: 92% vs. 64% *	5-year OP: 79% (CXB) vs. 56% (EBRT) *; CXB boost superior.
MONT-R TEM [30,31]	5-year DFS: 75.6% vs. 80.9% (NS); 5-year OS: 93.2% vs. 88.2% (NS)	pCR (TEM): 57.9% ypT0	TEM after cCR/near-cCR provides comparable survival with better function.
RAPIDO pCR	5-year OS after pCR: >90% (both arms)	-	Predictors of pCR: TNT (OR 2.70), CEA < 5 µg/L, tumor < 40 mm.
OPRA Grade	3-year DFS: 88% (CCR) vs. 69% (NCR) vs. 56% (ICR)	-	3-year OP: 77% (CCR) vs. 40% (NCR); three-tier response highly prognostic.
STELLAR LPLN [28]	3-year DFS in LPLN+: 51.7% vs. 66.2% (LPLN−)	-	LPLN+ remains a negative prognostic factor despite TNT.
CINTS-R (interim) [25,26]	Primary endpoint (2-year DrTF) not yet reported	-	ctDNA-guided stratification feasible; substantial discordance with clinical risk.
Polish II long-term [39]	8-year OS: 49% vs. 49% (NS); 8-year DFS: 43% vs. 41% (NS)	16% vs. 12% (NS)	Early OS benefit of SCRT-based TNT lost with longer follow-up; no difference in late complications.
MONT-R chemo [30,31]	3-year DFS: comparable (NS)	25.5% vs. 25.3% (NS)	Adding oxaliplatin to nCRT improved tumor regression (CAP 0–1: 58.6% vs. 46.8% *) but not survival.

* Statistically significant (*p* < 0.05). Abbreviations: MFS = metastasis-free survival; MPR = major pathological response; NS = not significant; other abbreviations as in Figure 2.

**Table 3:** Baseline risk characteristics of major TNT trials and de-escalation studies.

Trial	cT4 (%)	cN2 (%)	EMVI+ (%)	MRF+ (%)	Other High-Risk Features	Radiotherapy Backbone	pCR Rate (TNT Arm)	3-Year DFS/OS
High-risk LARC trials								
RAPIDO [11]	74%	86%	53%	~35–40%	-	SCRT (5 × 5 Gy)	28%	3-year DrTF 23.7% (improved)
PRODIGE 23 [12]	26%	~70%	Not reported	Not reported	-	LCRT (50.4 Gy)	27.5%	7-year OS 81.9% (improved)
STELLAR [13]	37%	74%	Not reported	Not reported	-	SCRT (5 × 5 Gy)	21.8%	3-year DFS 64.5% (NI)
TNTCRT [14]	Included (cT4a–b)	Included	Included	Included	Enlarged lateral nodes	LCRT (50–50.4 Gy)	27.5%	3-year DFS 77.0% (improved)
Polish II [39]	Majority cT4 or fixed cT3	Not specified	Not specified	Not specified	Locally recurrent (3%)	SCRT (5 × 5 Gy)	16%	8-year OS 49% (NS)
MONT-R chemo [30,31]	High-risk (cT4, cN2, EMVI+, MRF+)	Included	Included	Included	-	LCRT	25.5% (CapeOX)	3-year DFS comparable (NS)
Lower-risk/de-escalation trials								
PROSPECT [40]	0% (excluded cT4)	~75% (cN1–2)	Not specified	0% (CRM-negative)	Mid-upper rectal tumors	LCRT (selective)	21.4% (FOLFOX alone)	5-year DFS 80.8% (NI)
CONVERT [41]	0% (excluded cT4b, MRF+)	Included (cN2 allowed)	17–22%	0% (uninvolved MRF)	Distance 5–12 cm from AV	None (nCT alone)	~20%	3-year DFS 89.2% (comparable)
FOWARC [50]	~25–27% (cT4a–b)	~60–70%	Not reported	~31–35%	-	LCRT (in RT arms)	27.5% (mFOLFOX + RT)	10-year DFS 60–62% (NS)

**Table 4:** Toxicity, compliance, and patient-reported outcomes (updated).

Trial	Acute Grade ≥ 3 Toxicity (Pre-Operative)	Completion of Planned Pre-Operative Therapy	Notable Toxicity and Clinical Safety Observations
RAPIDO [45]	47.6% vs. 24.7% * (TNT vs. CRT)	84.6% vs. 90.0% *	No significant difference in global QoL, bowel function (LARS), or chronic toxicity at 3 years.
PRODIGE 23 [57]	46.9% vs. 35.6% * (TNT vs. CRT)	89.6% vs. 98.7% *	TNT transiently reduced QoL during CT; long-term QoL converged. Baseline physical function prognostic.
STELLAR [58]	26.5% vs. 12.6% * (TNT vs. CRT)	82.6% vs. 95.2% *	At 6-year follow-up, no clinically significant difference in global QoL or anal function (Wexner).
TNTCRT [14]	Thrombocytopenia (10.3% grade 3–4 in TNT arm)	High in both arms	TNT well tolerated; no significant difference in severe post-op morbidity.
PROSPECT [40]	41.0% vs. 22.6% * (FOLFOX vs. CRT)	89.5% vs. 84.3%	FOLFOX: higher neuropathy, fatigue, nausea; CRT: higher diarrhea. Long-term: CRT worse sexual function.
CONVERT [41]	Grade 2–4 long-term AEs: 16.0% vs. 26.3% *	~90% both arms	nCT significantly reduced proctitis (33.6% vs. 41.7%, *p* = 0.049) and long-term toxicity.
FOWARC 10-year [50]	Not reported in long-term update	Not reported	Long-term survival comparable; pCR associated with excellent outcomes (10-year OS 92.4%).
GRECCAR 4 [42]	Not reported	High	Response-adaptive strategy feasible; good responders avoided CRT toxicity.
UNION [18]	~45% vs. ~35%	~88% both arms	Adding camrelizumab increased irAEs (rash, thyroiditis) typically grade 1–2.
STELLAR II [20]	34.5% vs. 19.4% * (iTNT vs. TNT)	High both arms	Grade 3–4 irAEs: 5.5%; manageable.
SPRING-01 [21]	Grade 3–4: 33% vs. 35% (NS)	82% vs. 84%	Most common grade 3–4: thrombocytopenia (12% vs. 22%). No treatment-related deaths in iTNT arm.
PRECAM [22]	Grade 3: 2/32 (6.25%)	32/34 completed	Adverse events: tenesmus (78.1%), diarrhea (62.5%), leukopenia (40.6%); manageable.
NRG-GI002 [48]	Not reported in long-term	Not reported	No unexpected safety signals with pembrolizumab.
OPRA [43]	~38% (induction) vs. ~41% (consolidation)	~85%	No significant difference in late toxicity between sequences.
CAO/ARO/AIO-16 [49]	36% (during TNT)	90/91 RT; 82/88 CT	Sustained CCR patients had better bowel function (lower LARS/Wexner) at 18/36 months vs. immediate TME.
OPERA [24]	Not reported	High	CXB boost well tolerated; no increase in severe late toxicity.
MONT-R TEM [30,31]	Not applicable (post-nCRT)	-	TEM: significantly shorter operation time, less blood loss, shorter hospital stay, better sphincter function (Wexner 1 vs. 4, LARS 0 vs. 17).
PRORECT [23]	Acute ≥ G2 Diarrhea: 10% vs. 27% * (Proton vs. Photon)	Similar	Dosimetric comparison suggested proton therapy reduces acute GI toxicity.
MRI-LINAC LoRP [46]	Not reported	-	LoRP reduced treatment session duration by >20 min vs. fully adaptive; 92% of LoRP plans acceptable vs. 74% for couch shift.
Polish II long-term [39]	Acute toxicity lower in SCRT-based TNT	~85%	Late grade 3+ complications: 11% vs. 9% (NS); no difference in late toxicity.
MONT-R chemo [30,31]	Grade 3–4: 14.1% vs. 9.3% (NS)	~91%	CapeOX increased tumor regression (CAP 0–1) without significant increase in severe AEs.

* Statistically significant (*p* < 0.05). Abbreviations: as in original plus iTNT = immunotherapy plus TNT; LoRP = Library of Reference Plans; TEM = transanal endoscopic microsurgery; NS = not significant.

## Data Availability

No new data were created or analyzed in this study.

## Supplementary Materials

The following supporting information can be downloaded at: https://www.mdpi.com/article/10.3390/diseases14050182/s1, Table S1: PRISMA 2020 Checklist: Systematic Review.

## Abbreviations

The following abbreviations are used in this manuscript:

Adj CT	Adjuvant Chemotherapy
AE	Adverse Event
APC	Article Processing Charge (cover letter only)
APR	Abdominoperineal Resection
ASCO	American Society of Clinical Oncology
AV	Anal Verge
CAP	College of American Pathologists (tumour regression grading)
CapeOX/CAPOX	Capecitabine + Oxaliplatin (chemotherapy regimen)
cCR	Clinical Complete Response
CEA	Carcinoembryonic Antigen
CGA	Comprehensive Geriatric Assessment
CI	Confidence Interval
CINTS-R	ctDNA-guided neoadjuvant treatment strategy for locally advanced rectal cancer (trial)
CNA	Copy Number Alteration
CR	Complete Response
CRM	Circumferential Resection Margin
CRT	Chemoradiotherapy/Chemoradiation
CT	Chemotherapy or Computed Tomography
ctDNA	Circulating Tumour DNA
CTV	Clinical Target Volume
CXB	Contact X-ray Brachytherapy
DFS	Disease-Free Survival
dMMR	Deficient Mismatch Repair
DrTF	Disease-related Treatment Failure
DWI	Diffusion-Weighted Imaging
EBRT	External Beam Radiotherapy
ECOG	Eastern Cooperative Oncology Group
eCRF	Electronic Case Report Form
EDC	Electronic Data Capture
ELAPE	Extralevator Abdominoperineal Excision
EMBASE	Excerpta Medica dataBASE
EMVI	Extramural Vascular Invasion
ERUS	Endorectal Ultrasound
ESMO	European Society for Medical Oncology
EUS	Endoscopic Ultrasound
FOLFIRINOX	Folinic acid, Fluorouracil, Irinotecan, Oxaliplatin
FOLFOX	Folinic acid, Fluorouracil, Oxaliplatin
G2/G3/G4	Grade 2/Grade 3/Grade 4 (toxicity grading)
GI	Gastrointestinal
GTV	Gross Tumour Volume
Gy	Gray (unit of radiation dose)
HR	Hazard Ratio
ICI/ICIs	Immune Checkpoint Inhibitor(s)
ICR	Incomplete Response
IMRT	Intensity-Modulated Radiotherapy
irAE	Immune-related Adverse Event
ISR	Intersphincteric Resection
ITT	Intention-To-Treat
LARC	Locally Advanced Rectal Cancer
LARS	Low Anterior Resection Syndrome
LCRT	Long-Course Radiotherapy/Long-Course Chemoradiotherapy
LE	Local Excision
LLND	Lateral Lymph Node Dissection
LMIC	Low- and Middle-Income Countries
LoRP	Library of Reference Plans
LPLN	Lateral Pelvic Lymph Node(s)
LPLN+	Lateral Pelvic Lymph Node-Positive
LPLN−	Lateral Pelvic Lymph Node-Negative
LR	Locoregional Recurrence
LRRFS	Locoregional Recurrence-Free Survival
MeSH	Medical Subject Headings
mFOLFOX6	Modified FOLFOX regimen (6-drug variant)
MFS	Metastasis-Free Survival
MMR	Mismatch Repair
MPR	Major Pathological Response
MR	Magnetic Resonance
MRF	Mesorectal Fascia
MRF+	Mesorectal Fascia Involvement/Positive
MRF−	Mesorectal Fascia Uninvolved/Negative
MRI	Magnetic Resonance Imaging
MRI-LINAC	Magnetic Resonance Imaging-guided Linear Accelerator
MSI-H	Microsatellite Instability-High
MSS	Microsatellite Stable
mITT	Modified Intention-To-Treat
N+	Node-Positive
N0	Node-Negative
NACRT	Neoadjuvant Chemoradiotherapy
NAR	Neoadjuvant Rectal (score)
NCCN	National Comprehensive Cancer Network
nCRT	Neoadjuvant Chemoradiotherapy
NCR	Near-Complete Response
nCT	Neoadjuvant Chemotherapy
NGS	Next-Generation Sequencing
NI	Non-Inferiority
NK	Natural Killer (cells)
NOM	Non-Operative Management
NS	Not Significant
OAR	Organ at Risk
OP	Organ Preservation
OPS	Organ Preservation Strategies
OR	Odds Ratio
OS	Overall Survival
pCR	Pathological Complete Response
PD-1	Programmed Death-1
PD-L1	Programmed Death-Ligand 1
PET/CT	Positron Emission Tomography/Computed Tomography
pMMR	Proficient Mismatch Repair
PRISMA	Preferred Reporting Items for Systematic Reviews and Meta-Analyses
PRO	Patient-Reported Outcome
PROSPERO	International Prospective Register of Systematic Reviews
PTV	Planning Target Volume
QALY	Quality-Adjusted Life Year
QLQ-C30	Quality of Life Questionnaire—Core 30 (EORTC)
QoL	Quality of Life
RCT	Randomised Controlled Trial
RNA-seq	RNA Sequencing
RoB 2	Revised Cochrane Risk of Bias tool for randomised trials (version 2)
RT	Radiotherapy
SAE	Serious Adverse Event
SBRT	Stereotactic Body Radiotherapy
SCRT	Short-Course Radiotherapy
scRNA-seq	Single-Cell RNA Sequencing
SIB	Simultaneous Integrated Boost
SNI	Selective Nodal Irradiation
SNV	Single-Nucleotide Variant
TaTME	Transanal Total Mesorectal Excision
TCGA	The Cancer Genome Atlas
TEM	Transanal Endoscopic Microsurgery
TIL	Tumour-Infiltrating Lymphocyte
TMB	Tumour Mutational Burden
TME	Total Mesorectal Excision
TNT	Total Neoadjuvant Therapy
TPS	Tumour Proportion Score
TRG	Tumour Regression Grade
VAF	Variant Allele Frequency
W&W	Watch-and-Wait
XELOX	Capecitabine + Oxaliplatin (same as CAPOX)
yp	Pathological stage after neoadjuvant therapy
