# Supplementary material for: Total Neoadjuvant Therapy, Organ Preservation and Beyond: A State-of-the-Art Systematic Review and Critical Appraisal of Locally Advanced Rectal Cancer Management
Source: Diseases. 2026 May 21;14(5):182. doi: 10.3390/diseases14050182 (PMC13205331; doi:10.3390/diseases14050182)
Supplement: Supplementary file 1 [file diseases-14-00182-s001.zip › diseases-4292939-supplementary.pdf]

PRISMA 2020 Checklist: Systematic Review

Section / Topic	Item #	Checklist item	Location in manuscript (page/section)	Reported?
**TITLE**				
Title	1	Identify the report as a systematic review.	Title page: "A state-of-the-art systematic review and critical appraisal"	✓
**ABSTRACT**				
Abstract	2	Structured summary (background, methods, results, discussion/conclusions).	Abstract (lines 31–58)	✓
**INTRODUCTION**				
Rationale	3	Describe the rationale for the review.	Introduction, paragraphs 1–3	✓
Objectives	4	Provide an explicit statement of the objectives.	End of Introduction (lines 113–120)	✓
**METHODS**				
Eligibility criteria	5	Specify inclusion and exclusion criteria.	Section 2.2	✓
Information sources	6	Specify databases, dates, and other sources.	Section 2.1	✓
Search strategy	7	Present full search strategy (at least one database).	Section 2.1 (brief) + Supplementary File S2 (full PubMed strategy) - added per PRISMA requirement	✓ (with supplement)

Section / Topic	Item #	Checklist item	Location in manuscript (page/section)	Reported?
Selection process	8	Specify methods for selecting studies.	Section 2.2	✓
Data collection process	9	Specify methods for data extraction.	Section 2.4	✓
Data items	10	List all outcomes and variables extracted.	Section 2.4 + Table 1 and Table 3 footnotes	✓
Study risk of bias assessment	11	Specify method for assessing risk of bias (e.g., RoB 2).	Section 2.3 (RoB 2 mentioned) and Section 3.1.1 (results presented)	✓
Effect measures	12	Specify effect measures (e.g., HR, OR, pCR rate).	Results section (Tables 2, 3, 4)	✓
Synthesis methods	13a	Describe methods for synthesis (narrative).	Section 2.5	✓
	13b	Specify any software or tools used.	Not applicable (narrative synthesis)	✓
	13c	Describe methods for handling heterogeneity.	Section 2.5	✓
	13d	Not applicable (no meta-analysis).	-	✓
	13e	Describe methods for certainty assessment (e.g., GRADE).	Not performed - stated as limitation in Section 4.8.1	✓ (limitation)
Reporting bias assessment	14	Describe methods for assessing reporting bias.	Not formally assessed - stated as limitation in Section 4.8.1	✓ (limitation)
Certainty assessment	15	Describe methods for assessing certainty in body of evidence.	Not performed - stated as limitation in Section 4.8.1	✓ (limitation)

Section / Topic	Item #	Checklist item	Location in manuscript (page/section)	Reported?
**RESULTS**				
Study selection	16a	Describe results of the search and selection process (PRISMA flow diagram).	Section 3.1.2 + Figure 1	✓
	16b	Include reasons for exclusions.	Figure 1 legend	✓
Study characteristics	17	Describe characteristics of included studies (e.g., sample size, interventions).	Table 1 (pages 8–15)	✓
Risk of bias	18	Present assessments of risk of bias.	Section 3.1.1 (summary: all RCTs low risk of bias; single-arm studies not assessed)	✓
Results of individual studies	19	Present results for each included study (e.g., effect estimates).	Tables 1, 3, 4, 5	✓
Synthesis of results	20a	Summarize findings for each main outcome.	Sections 3.2 – 3.10	✓
	20b	Not applicable (no meta-analysis).	-	✓
	20c	Not applicable (no meta-analysis).	-	✓
Reporting biases	21	Present assessments of reporting bias.	Not done – stated as limitation in Section 4.8.1	✓ (limitation)
Certainty of evidence	22	Present assessments of certainty (e.g., GRADE).	Not done – stated as limitation in Section 4.8.1	✓ (limitation)
**DISCUSSION**				

Section / Topic	Item #	Checklist item	Location in manuscript (page/section)	Reported?
Discussion	23a	Provide interpretation in context of other evidence.	Section 4	✓
	23b	Discuss limitations of the review.	Section 4.8.1 (explicit limitations)	✓
	23c	Discuss implications for practice and research.	Section 4.8.2	✓
**OTHER INFORMATION**				
Registration	24a	Provide registration information (PROSPERO ID).	Section 2.4 (CRD420251252675)	✓
	24b	Indicate where protocol can be accessed.	Section 2.4	✓
Conflict of interest	25	Declare any competing interests.	End of manuscript (conflict of interest statement)	✓
Funding	26	Describe sources of funding.	Funding statement (end of manuscript)	✓
Data availability	27	State whether data are available.	Data Availability Statement (end of manuscript)	✓
Supporting information	28	List supplementary files (PRISMA checklist).	End of manuscript (Supplementary File S1)	✓
